# Ethnic and diet-related differences in the healthy infant microbiome

**DOI:** 10.1186/s13073-017-0421-5

**Published:** 2017-03-29

**Authors:** Jennifer C. Stearns, Michael A. Zulyniak, Russell J. de Souza, Natalie C. Campbell, Michelle Fontes, Mateen Shaikh, Malcolm R. Sears, Allan B. Becker, Piushkumar J. Mandhane, Padmaja Subbarao, Stuart E. Turvey, Milan Gupta, Joseph Beyene, Michael G. Surette, Sonia S. Anand, for the NutriGen Alliance

**Affiliations:** 1 Department of Medicine, McMaster University, Hamilton, ON Canada; 2 Farncombe Family Digestive Health Research Institute, McMaster University, Hamilton, ON Canada; 3 Department of Health Research Methods, Evidence, and Impact, McMaster University, Hamilton, ON Canada; 4 Department of Immunology, Faculty of Medicine, University of Manitoba, Winnipeg, Manitoba Canada; 5 Department of Pediatrics, Faculty of Medicine and Dentistry, University of Alberta, Edmonton, Alberta Canada; 6 Hospital for Sick Children & Department of Paediatrics, University of Toronto, Toronto, ON Canada; 7 BC Children’s Hospital and Child and Family Research Institute, Department of Paediatrics, Faculty of Medicine, University of British Columbia, Vancouver, British Columbia Canada; 8 Population Health Research Institute, Hamilton Health Sciences and McMaster University, Hamilton, Ontario Canada

**Keywords:** Infant gut microbiome, Ethnicity, Breastfeeding, Diet

## Abstract

**Background:**

The infant gut is rapidly colonized by microorganisms soon after birth, and the composition of the microbiota is dynamic in the first year of life. Although a stable microbiome may not be established until 1 to 3 years after birth, the infant gut microbiota appears to be an important predictor of health outcomes in later life.

**Methods:**

We obtained stool at one year of age from 173 white Caucasian and 182 South Asian infants from two Canadian birth cohorts to gain insight into how maternal and early infancy exposures influence the development of the gut microbiota. We investigated whether the infant gut microbiota differed by ethnicity (referring to groups of people who have certain racial, cultural, religious, or other traits in common) and by breastfeeding status, while accounting for variations in maternal and infant exposures (such as maternal antibiotic use, gestational diabetes, vegetarianism, infant milk diet, time of introduction of solid food, infant birth weight, and weight gain in the first year).

**Results:**

We demonstrate that ethnicity and infant feeding practices independently influence the infant gut microbiome at 1 year, and that ethnic differences can be mapped to alpha diversity as well as a higher abundance of lactic acid bacteria in South Asians and a higher abundance of genera within the order *Clostridiales* in white Caucasians.

**Conclusions:**

The infant gut microbiome is influenced by ethnicity and breastfeeding in the first year of life. Ethnic differences in the gut microbiome may reflect maternal/infant dietary differences and whether these differences are associated with future cardiometabolic outcomes can only be determined after prospective follow-up.

**Electronic supplementary material:**

The online version of this article (doi:10.1186/s13073-017-0421-5) contains supplementary material, which is available to authorized users.

## Background

The developing gastrointestinal microbiota in the first years of life is important for immune function, nutrient metabolism and protection from pathogens [1–3]. Microbial colonization of the infant gut proceeds through infancy and establishment of an adult-like microbiome is estimated to occur within the first 3 years [4]. Identifying factors that shape the gut microbiome is currently an active area of research and early evidence suggests that host genetics [5] and early life exposures, including delivery method, antibiotics [6, 7], and diet, influence the infant gut microbiome [8, 9]. In addition to these established roles, the gut microbiota is emerging as a potentially important contributor to the development of non-communicable diseases (NCDs), having been associated with conditions such as obesity [10, 11], type 2 diabetes [12, 13], allergy and atopy [14], inflammatory bowel disease [15], and the development of colon cancer [16]. The influence of the infant microbiome on the development of these conditions is of great clinical and economic interest as rates of NCDs in adults are increasing globally and by 2030 are predicted to account for 89% of all deaths in high income countries [17].

South Asians are people whose ancestors originate from the Indian subcontinent and they have among the highest rates of type 2 diabetes and premature cardiovascular disease (CVD) in the world. CVD risk factors, including adiposity, type 2 diabetes, and dyslipidemia, are higher among South Asians compared to white Caucasians of the same BMI [18]. There is preliminary evidence that gut microbial composition in adults and children varies by age [4, 19], dietary intake [20, 21], ethnicity, geography [4, 22], and adoption of western lifestyles [19, 23]. Bacterial richness has been shown to increase with age and to be lower in residents of the United States compared with other populations [4]. Core bacterial metabolic genes varied between these populations as well; however, the underlying reasons for ethnic and geographic differences in the microbiome have not been characterized. In this paper we investigate the associations of ethnicity and early life exposures with the gut microbiome among 1-year-old infants born and living in Canada while accounting for a diverse set of covariates that represent dietary differences as well as other exposures throughout infancy. This study explores the effect of ethnicity separately from region and provides a preliminary look at effects of ethnicity on the gut microbiota in early life.

## Methods

### Cohorts

Participants from two prospective Canadian birth cohorts were included in this gut microbiome substudy. The Canadian Healthy Infant Longitudinal Development study (CHILD) enrolled 3624 mainly white Caucasian mother–child pairs and most fathers from four Canadian centers (Vancouver, BC; Edmonton, AB; Winnipeg/Winkler-Morden, MB; and Toronto, ON) to investigate the root causes of allergy and asthma, including genetic and environmental triggers, and the ways in which they interact [24–26]. In this analysis, ethnicity refers to groups of people who have certain racial, cultural, religious, or other traits in common, whereas race refers to a person’s physical characteristics, such as bone structure, or skin, hair, or eye color [27, 28]. In the CHILD cohort, white Caucasian ancestry was confirmed by participants’ response to the question “To which ethnic or cultural group did your parents belong?” The South Asian Birth Cohort (START-Canada) enrolled 1012 South Asian mother–child pairs from the Brampton and Peel Region of Ontario to investigate the influence of diverse environmental exposures and genetics on early life adiposity, growth trajectory, and cardiometabolic factors [29]. South Asian ethnicity was verified by the mother’s self-report of her and the father’s, and their parents’, ancestral origin being from India, Pakistan, Sri Lanka, or Bangladesh.

Harmonization of clinical data across cohorts was done by extracting them with the same definitions, where possible. When questions were not identical, we worked to extract the data from each cohort in such a way as to satisfy the same definition. Gestational diabetes mellitus was defined as having diabetes on the birth chart but no diabetes prior to pregnancy. A child was considered to have had formula in the first year if formula use was recorded at any time in the first year (from several questionnaires). In both cohorts, timing of infant weighing at 1 year was typically performed on the same day as 1-year stool collection (r^2^ > 0.93; median = 0 days; 95% confidence interval 0 to 2 days).

In this gut microbiome substudy, 1-year fecal samples from 173 white Caucasian infants in CHILD and 182 South Asian infants in START were used for the main analysis. An additional 77 samples from the CHILD cohort, from infants who are not white Caucasian, were used to explore trends found in the main analysis. For both cohorts, the collection of 1-year fecal samples was scheduled with the mother in advance. Stool collection was taken from a regular diaper in START and a specially lined diaper in CHILD [24]. Mothers were instructed to record the time and date of the stool sample and place it in a sterile bag in the refrigerator for their scheduled appointment with the research nurse. Upon arrival, the nurse used depyrogenized stainless steel spatulas to divide the sample between four pre-labeled cryovials. The cryovials were then transported to the lab in a cooler, weighed, and stored at −80 °C or liquid nitrogen. START samples were stored at 4 °C for 2–4 h prior to freezing whereas CHILD samples were stored at 4 °C for an average of 14 ± 12 h.

### DNA extraction, 16S rRNA gene sequencing, and analysis

DNA was extracted with a custom DNA extraction protocol described in [30]. Briefly, 100–200 mg of stool was added to 2.8 mm and 0.1 mm glass beads (MoBio Laboratories Inc., Carlsbad, CA, USA) along with 800 μl of 200 mM sodium phosphate monobasic (pH 8) and 100 μl guanidinium thiocyanate EDTA N-lauroylsarkosine buffer (50.8 mM guanidine thiocyanate, 100 mM ethylenediaminetetraacetic acid, and 34 mM N-lauroylsarcosine). These were then homogenized in a PowerLyzer 24 Bench Top Homogenizer (MoBio Laboratories Inc.) for 3 min at 3000 RPM. Next, two enzymatic lysis steps were performed. First, the sample was incubated with 50 μl of 100 mg/ml lysozyme, 500 U mutanolysin, and 10 μl of 10 mg/ml RNase for 1 h at 37 °C. Next, the sample was incubated with 25 μl 25% sodium dodecyl sulphate, 25 μl of 20 mg/ml Proteinase K, and 62.5 μl of 5 M NaCl at 65 °C for 1 h. Next, debris was pelleted in a tabletop centrifuge at maximum speed for 5 min and the supernatant added to 900 μl of phenol:chloroform:isoamyl alcohol (25:24:1). The sample was then vortexed and centrifuged at maximum speed in a tabletop centrifuge for 10 min. The aqueous phase was removed and the sample run through the Clean and Concentrator-25 column (Zymo Research, Irvine, CA, USA) according to kit directions except for elution, which was done with 50 μl of ultrapure water and allowed to sit for 5 min before elution. The DNA was quantified using a Nanodrop 2000c Spectrophotometer [30]. Amplification of the bacterial 16S rRNA gene v3 region (150 bp) tags was performed as previously described [31] with the following changes: 5 pmol of primer, 200 μM of each dNTP, 1.5 mM MgCl_2_, 2 μl of 10 mg/ml bovine serum albumin, and 1.25 U Taq polymerase (Life Technologies, Carlsbad, CA, USA) were used in a 50 μl reaction volume. The PCR program used was as follows: 94 °C for 2 min followed by 30 cycles of 94 °C for 30 s, 50 °C for 30 s, and 72 °C for 30 s, then a final extension step at 72 °C for 10 min. DNA extraction and PCR amplification of 16S rRNA gene v3 libraries were found to be reproducible using a set of five samples from each cohort (total of ten samples) that were extracted in triplicate (29 extractions since one extraction failed) and a subset of three extractions from each cohort amplified in triplicate for a total of 41 datasets (Additional file 1: Figure S1).

Illumina libraries were sequenced in the McMaster Genomics Facility with 250-bp sequencing in the forward and reverse directions on the Illumina MiSeq instrument. Custom, in-house Perl scripts were used to process Illumina sequences as previously described [32]. Briefly, after sequence trimming and alignment, operational taxonomic units (OTU) were clustered using AbundantOTU+ [33] with a threshold of 97%. Chimera checking was not done since we have shown that amplification of the short V3 region of the 16S rRNA gene leads to very few genuine chimeric sequences [34]. Taxonomy for the representative sequence of each OTU was assigned using the Ribosomal Database Project classifier [35] with a minimum confidence cutoff of 0.8 against the Greengenes (2013 release) reference database [36]. All OTUs classified as “Root:Other” (comprising 0.03% of the total reads sequenced) were then excluded as was one sample with <500 sequenced reads; however, singleton OTUs were not excluded. This resulted in a total of 41.4 million reads with a minimum of 2.0 × 10^3^, maximum of 4.3 × 10^5^, and a median of 9.0 × 10^4^ reads per sample.

Bacterial community richness and diversity (alpha diversity) were calculated using the estimated species richness and Shannon diversity functions with the vegan package in R [37], using OTU abundances. Differences between bacterial communities in each sample (beta diversity) were quantified using the Bray–Curtis dissimilarity measure on relative abundance values of all bacterial genera and principal coordinate analysis was also done using the vegan package or the phyloseq package [38] in R.

### Statistical Analysis

Simple linear regression was used to determine the effect of ethnicity and breastfeeding on alpha diversity estimates. Permutational multivariate analysis of variance on Bray–Curtis dissimilarities of genus level relative abundances, done with the adonis function from the vegan package in R [37], was used to examine bacterial community differences associated with ethnicity after adjustment for potential covariates of ethnicity–microbiome associations.

Candidate covariates in the multivariable model were informed by the existing literature and assessed formally in univariable models against microbiome diversity (i.e., years mother lived in Canada, breastfeeding at time of collection, time since weaning, formula and cow’s milk use in the first year, time of introduction of solid foods, infant weight gain in the first year, birth weight, infant age at stool collection, and mode of delivery, gestational diabetes, mother’s antibiotic use during pregnancy and labor, and mother’s vegetarian status). Next, the candidate variables chosen above were used to separately predict dissimilarities with the same method as above. Those with *p* < 0.10 were subjected to a forward stepwise procedure. We then added the most significant covariates into the model in order of the proportion of variance explained, and stopped when the next most significant covariate was above the 0.05 threshold.

The association between genus level abundances and ethnicity and/or breastfeeding was determined through a multivariate algorithm adjusting for significant covariates performed with the Maaslin package in R [39, 40]. Briefly, covariates found to be significant (*p* < 0.05) predictors of the microbiome (described above) were included into a multivariate boosted, additive general linear model between covariate data and bacterial genus level abundances. *P* values were adjusted for multiple testing with the false discovery rate, reported as q values, and q < 0.05 was considered significant. Genera with a coefficient of variation >0.001 were included in Additional file 2: Table S1.

## Results

Table 1 shows the baseline demographic and anthropometric characteristics of the mothers and infants selected from CHILD (white Caucasians only) and START. Briefly, South Asian mothers lived in Canada for an average of 8 years versus a lifetime for white Caucasian mothers. Furthermore, South Asian mothers were younger, more likely to be vegetarian (34% versus 2%, *p* < 0.001), and to be diagnosed with gestational diabetes during pregnancy (14% versus 4%, *p* < 0.001) compared to white Caucasian mothers. There were no significant differences in the rates of Caesarian section between ethnic groups (18% in South Asian versus 15% in white Caucasian); however, white Caucasian mothers were more likely to receive antibiotics during pregnancy (8% versus 0.5%, *p* < 0.001) and South Asian mothers were more likely to receive antibiotics during labor (43% versus 34%, *p* < 0.05). South Asian infants were born earlier (39.1 weeks versus 39.5 weeks, *p* < 0.05), had lower birth weight (3.3 kg versus 3.5 kg, *p* < 0.001), and gained more weight in the first year of life (7.1 kg gained versus 6.4 kg gained, *p* < 0.001) than did white Caucasian infants. While both white Caucasian and South Asian mothers reported that they breastfed their infants at some point during the first year (97.1% versus 94.4%), a greater proportion of South Asian infants were still breastfeeding at the time of 1-year stool sample collection (43% versus 32%, *p* < 0.05). Additionally, there was more formula use during the first year (77% versus 65%, *p* < 0.001) and earlier introduction of solid food among South Asians (88% versus 50% from 3 to 6 months, 9.4% versus 40% from 6 to 9 months, *p* < 0.001). We suspect that more South Asian infant diets were vegetarian on account of the greater proportion of their mothers who identified as vegetarian (34% versus 2%, *p* < 0.001). Furthermore, there was no difference in age at time of stool collection (*p* = 0.39) between white Caucasians (12.3 ± 1.71 months) and South Asians (12.4 ± 1.69 months; Table 1).

**Table 1:** Mother and infant characteristics

	White Caucasians	South Asians	*P* value
Maternal characteristics^a^			
Number per group	173	182	
Maternal age in years (SD)	31.9 (4.13)	30.5 (4.03)	0.002^#^
Maternal height (cm)	166.3 (6.54)^c^	161.5 (6.54)^b^	<0.001^#^
Maternal pre-pregnancy BMI (if available)	24.6 (4.65)^c^	24.2 (4.51)	>0.05^#^
Maternal weight gain	15.2 (5.73)^f^	15.3 (8.99)^b^	>0.05^#^
Years mother lived in Canada (SD)	29.5 (7.71)^b^	7.7 (5.96)	<0.001^#^
Vegetarian status of mother N (%)^‡^	4 (2.31%)	62 (34.07%)^b^	<0.001*
Gestational diabetes N (%)	7 (4.05%)^b^	26 (14.29%)^b^	<0.001*
Mode of delivery N (%)			
Vaginal	123 (82.00%)^c^	121 (78.57%)	>0.05*
C-Section	27 (18.00%)^c^	33 (21.43%)	
Antibiotics during pregnancy			
Yes	14 (8.09%)	1 (0.55%)	<0.001*
No	159 (91.91%)	181 (99.45%)	
Antibiotics during labor			
Yes	59 (38.06%)^d^	79 (45.40%)^b^	0.036*
No	96 (61.94%)^d^	95 (54.60%)^b^	
Infant covariates^a^			
Currently breastfeeding at sample collection N			
Yes	56 (36.13%)^b^	78 (43.33%)^b^	0.036*
No	99 (63.87%)^b^	102 (56.67%)^b^	
Breastfed in the first year			
Yes	167 (97.1%)^b^	170 (94.4%)^b^	>0.05*
No	5 (2.9%)^b^	10 (5.5%)^b^	
Time since weaning in months (SD)	5.6 (4.02)^f^	7.1 (5.85)^f^	0.045^#^
Currently using cow’s milk			
Yes	68 (57.63%)^e^	121 (67.98%)^b^	0.019*
No	50 (42.37%)^e^	57 (32.02%)^b^	
Currently using formula			
Yes	47 (31.13%)^d^	72 (39.56%)	0.026*
No	104 (68.87%)^d^	110 (60.44%)	
Formula in the first year			
Yes	99 (63.06%)^c^	154 (90.06%)^c^	<0.001*
No	58 (36.94%)^c^	17 (9.94%)^c^	
Time in months of introduction of solids			
0–3	6 (4.03%)^d^	2 (1.11%)^b^	<0.001^§^
3–6	75 (50.34%)^d^	159 (88.33%)^b^	
6–9	66 (44.30%)^d^	17 (9.44%)^b^	
9–12	2 (1.34%)^d^	2 (1.11%)^b^	
Birth weight in kg (SD)	3.5 (0.47)^c^	3.3 (0.48)	<0.001^#^
Weight gain in the first year in kg (SD)	6.4 (1.21)^c^	7.1 (1.31)	<0.001^#^
Age of infant at time of sample collection in months (SD)	12.3 (1.71)	12.4 (1.69)	0.39^#^
Gestational age in weeks (SD)	39.5 (1.33)^b^	39.1 (1.36)	0.006^#^

^a^where there is no superscript there was no missing data

^b^Less than 5.00% data missing

^c^Less than 10.00% data missing

^d^10–20% data missing

^e^32% data missing

^f^40–50% data missing

*Fischer’s exact test

^§^Cochrane Armitage trend test

^‡^Maternal vegetarian status was used as a surrogate for infant diet exposure

^#^ *T*-test﻿

### Abundance of microorganisms within all samples

**Fig. 1:**
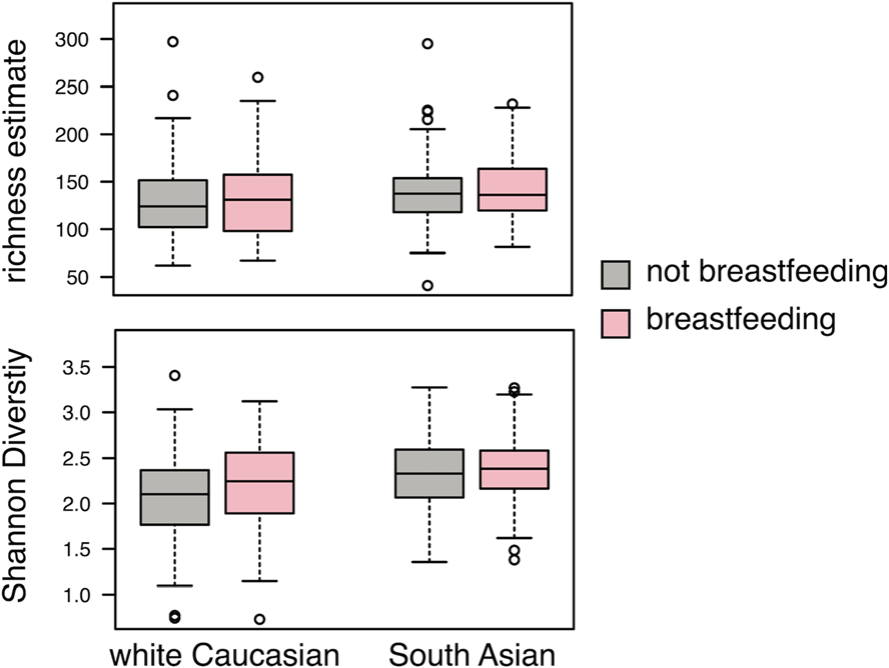
Alpha diversity measures within white Caucasians and South Asians, split by breastfeeding status at the time of sample collection. *Whiskers* extend to the most extreme data values up to 1.5× the interquartile range; data outside this range are shown as *circles*

The v3 region of 16S rRNA genes was profiled from 355 participant stool samples collected at 1 year of age, 173 white Caucasians from the CHILD cohort and 182 from the START cohort. The range of alpha diversity estimates for each ethnicity separated by current breastfeeding at the time of sampling is illustrated in Fig. 1. Using simple linear regression, species richness estimates were found to be significantly affected by ethnicity after taking into account breastfeeding at time of collection (*p* < 0.05). Shannon diversity was significantly affected by ethnicity, taking into account breastfeeding at time of collection (*p* < 0.001), and likewise breastfeeding at time of collection within each ethnicity significantly affected Shannon diversity (*p* < 0.05). Further, when START samples, all collected within the Brampton/Peel region of Ontario Canada, were compared with each study center within the CHILD cohort (Vancouver, Edmonton, Toronto, and Winnipeg/Winkler-Morden) only Winnipeg/Winkler-Morden, MB had significantly lower species richness estimates (*p* < 0.05; Additional file 1: Figure S2). Although there was variability in Shannon diversity estimates across sample sites for CHILD, all sites were found to have significantly lower diversity than the START samples (*p* < 0.05; Additional file 1: Figure S2), while accounting for current breastfeeding. By including sample sites into the regression model the effect of current breastfeeding on Shannon diversity was no longer significant (*p* = 0.054).

Differences in the relative abundance of the dominant bacterial genera are presented in Additional file 1: Figure S3, broken down by ethnic group and breastfeeding status. Heterogeneity of samples can be seen in Additional file 1: Figure S3 as well as differences in genus level microbobial profiles between ethnic groups and breastfeeding status, differences that are explored in detail below.

**Fig. 2:**
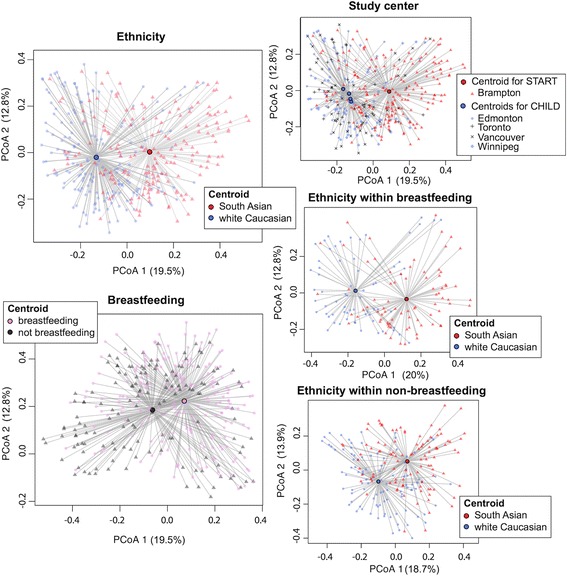
Principal coordinate analyses (*PCoA*) of Bray–Curtis dissimilarities. Centroids for ethnicity, breastfeeding status at time of collection, and study center are shown as *circles* with *lines* radiating to samples

Principal coordinate analysis of Bray–Curtis dissimilarities illustrates between-community differences in samples from white Caucasians and South Asian infants. Variation in the gut microbiome across geography has been observed in studies involving adults [41]; however, in our study the effect of ethnicity was larger than the effect of geographic location (Fig. 2) shown as the separation of the centroid for samples from South Asians from the centroids of samples from white Caucasians from all study centers. Also evident from the principal coordinate analysis, breastfeeding at time of collection affected the gut microbial profiles, although when stratified by currently breastfed and not currently breastfed infants, the strong effect of ethnicity persisted (Fig. 2). Several studies have found the infant gut microbiome to vary between infants born by Caesarean section and those born vaginally with the effect diminishing with age. Here, delivery method was not found to be a significant predictor of the structure of the gut microbiome in 1-year-old infants (Additional file 1: Figure S4). This may be because differences were no longer strong enough to be detected or because members of the phylum Bacteroidetes, often missing from the gut microbiome in Caesarean section delivered infants, were not abundant in our vaginally born infants (Additional file 1: Figure S2).

### Association between ethnicity, milk diet, and solid food diet

In addition to ethnicity, 13 potential covariates were also associated with the microbiome in univariable regression analysis. These included mother’s years living in Canada, infant age, breastfeeding status at time of collection, time since weaning, vegetarian status, timing of introduction of solid foods, birth weight, infant weight gain in the first year, antibiotics during pregnancy, antibiotics during labor, formula use in the first year, formula use at collection, and cow’s milk in the first year (all *p* < 0.10; Table 2). We entered this set of covariates into a forward stepwise regression model to determine which factors remained significant and independently influenced the gut microbiome. Only ethnicity (*p* < 0.001), breastfeeding status (*p* < 0.001), infant age at stool collection (*p* < 0.01), and weight gain in the first year (*p* < 0.01) remained independently associated with the gut microbiome as a whole.

**Table 2:** Univariable and multivariable permutational analysis of variance using Bray–Curtis dissimilarity matrices

Predictor variable	Univariable			Multivariable		
	F-Model	R^2^	Pr(>F)	F-Model	R^2^	Pr(>F)
Ethnicity	32.61	0.084	0.001	32.21	0.084	0.001
Years in Canada	24.57	0.066	0.001	NA^a^		
Infant age at time of sample collection	3.79	0.011	0.003	3.75	0.01	0.002
Breastfeeding at 1 year	14.28	0.040	0.001	12.75	0.033	0.001
Time since weaning	5.46	0.018	0.001	NA		
Delivery mode	0.82	0.005	0.64	NA		
Vegetarian status	10.45	0.029	0.001	NA		
Gestational diabetes	1.49	0.004	0.16	NA		
Antibiotics during pregnancy	2.07	0.006	0.04	NA		
Antibiotics during labor	2.56	0.008	0.02	NA		
Time of introduction of solid food	1.80	0.016	0.02	NA		
Birth weight	3.09	0.009	0.006	NA		
Infant weight gain in the first year	5.09	0.014	0.001	3.08	0.008	0.01
Formula at collection	1.05	0.004	0.36	NA		
Formula in the first year	2.35	0.007	0.03	NA		
Cow’s milk in the first year	1.85	0.006	0.06	NA		

Multivariable model chosen by forward stepwise regression

^a^Not included as highly collinear with ethnicity

*NA* not applicable

There was no statistically significant multiplicative interaction between ethnicity and breastfeeding (*p* = 0.23). Nevertheless, we acknowledge that such tests may be underpowered, and thus the results were also stratified by ethnicity and breastfeeding status in order to examine trends. Forward stepwise regression was conducted within white Caucasians and separately within South Asians (Table 3). This revealed that breastfeeding (*p* < 0.01) and infant age (*p* < 0.05) were independently associated with differences in the microbiome within each ethnic group, while antibiotic use during labor (*p* < 0.05) and weight gain in the first year (*p* < 0.05) remained independently associated with differences in the microbiome only in white Caucasians. Forward stepwise regression was also conducted separately within infants breastfed and not breastfed at the time of collection (Table 4), which indicated that ethnicity (*p* < 0.01) and the infant age (*p* < 0.05) remained independently associated with differences in the gut microbiome in both groups.

**Table 3:** Subgroup analysis based on ethnicity. Permutational analysis of variance using Bray-Curtis dissimilarity matrices

Predictor variable	Univariable			Multivariable		
	F-Model	R^2^	Pr(>F)	F-Model	R^2^	Pr(>F)
White Caucasians						
Breastfeeding	6.52	0.038	0.001	4.18	0.03	0.001
Time since weaning	2.81	0.020	0.007	NA		
Age at time of sample collection	2.89	0.017	0.007	2.5	0.015	0.02
Delivery mode	0.98	0.012	0.48	NA		
Antibiotics during pregnancy	0.52	0.003	0.86	NA		
Antibiotics during labor	2.45	0.016	0.03	NA		
Vegetarian status	2.33	0.013	0.02	NA		
Gestational diabetes	0.85	0.005	0.52	NA		
Time of introduction of solid food	1.12	0.023	0.32	NA		
Formula at collection	1.23	0.009	0.26	NA		
Formula in the first year	2.51	0.016	0.01	NA		
Cow’s milk in the first year	1.29	0.01	0.23	NA		
Weight gain in the first year	2.23	0.014	0.04	2.8	0.017	0.01
Birth weight	0.35	0.002	0.95	NA		
South Asians						
Breastfeeding	8.79	0.047	0.001	8.88	0.047	0.001
Age at time of collection	3.75	0.020	0.003	2.84	0.015	0.008
Time since weaning	5.23	0.030	0.001	NA		
Delivery mode	1.10	0.012	0.34	NA		
Antibiotics during pregnancy	0.52	0.003	0.85	NA		
Antibiotics during labor	2.45	0.016	0.02	NA		
Vegetarian status	1.32	0.007	0.22	NA		
Gestational diabetes	0.48	0.003	0.88	NA		
Years lived in Canada	0.92	0.005	0.46	NA		
Time of introduction of solid food	1.35	0.022	0.14	NA		
Formula at collection	2.07	0.015	0.05	NA		
Formula in the first year	1.95	0.01	0.06	NA		
Cow’s milk in the first year	2.24	0.012	0.04	NA		
Weight gain in the first year	1.40	0.008	0.19	NA		
Birth weight	1.27	0.007	0.24	NA		

Permutational analysis of variance using Bray-Curtis dissimilarity matrices

*NA* not applicable

**Table 4:** Subgroup analysis of breastfed and not currently breastfed children at time of collection. Permutational analysis of variance using Bray-Curtis dissimilarity matrices

	F-Model	R^2^	Pr(>F)	F-Model	R^2^	Pr(>F)
Currently breastfed						
Ethnicity	15.43	0.104	0.001	18.01	0.112	0.001
Age	1.78	0.014	0.13	2.43	0.015	0.02
Delivery mode	1.06	0.015	0.38	NA		
Antibiotics during pregnancy	1.32	0.009	0.21	NA		
Antibiotics during labor	3.16	0.023	0.009	NA		
Vegetarian status	5.69	0.040	0.001	2.17	0.01	0.04
Gestational diabetes	1.05	0.007	0.36	NA		
Years lived in Canada	10.39	0.070	0.001	NA		
Time of introduction of solid food	4.88	0.066	0.001	NA		
Formula at collection	0.72	0.007	0.65	NA		
Formula in the first year	2.81	0.02	0.009	NA		
Cow’s milk in the first year	0.94	0.008	0.43	NA		
Weight gain in the first year	7.26	0.05	0.001	3.55	0.02	0.008
Birth weight	2.74	0.019	0.01	NA		
Not currently breastfed						
Ethnicity	16.10	0.074	0.001	11.54	0.06	0.001
Infant age	2.46	0.012	0.02	2.14	0.01	0.03
Time since weaning	3.20	0.019	0.002	NA		
Delivery mode	1.03	0.010	0.40	NA		
Antibiotics during pregnancy	2.10	0.010	0.049	NA		
Antibiotics during labor	1.54	0.008	0.15	NA		
Vegetarian status	3.76	0.019	0.002	NA		
Gestational diabetes	1.27	0.006	0.22	NA		
Years in Canada	12.79	0.062	0.001	NA		
Time of introduction of solid food	2.25	0.035	0.006	NA		
Formula at collection	1.63	0.009	0.11	NA		
Formula in the first year	2.48	0.013	0.01	NA		
Cow’s milk in the first year	0.88	0.005	0.55	NA		
Weight gain in the first year	2.25	0.01	0.03	NA		
Birth weight	2.20	0.011	0.03	NA		

*NA* not applicable

### Differentially abundant genera within each group

**Fig. 3:**
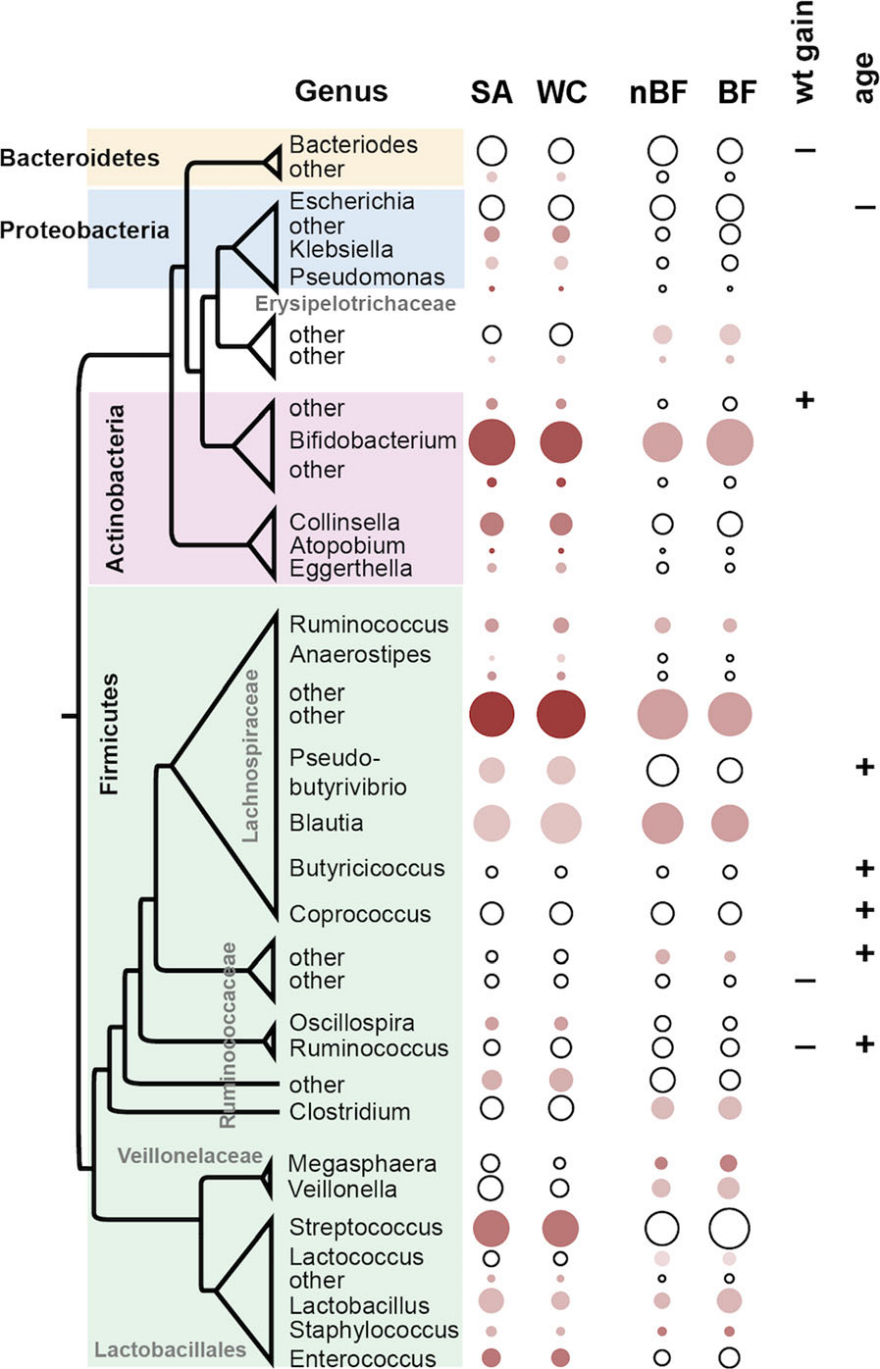
Genera differentially associated with ethnicity (white Caucasian (*WC*) and South Asian (*SA*)), breastfeeding (breastfeeding (*BF*) and not breastfeeding (*nBF*)), infant age, or infant weight gain in the first year (*wt gain*), through the multivariate boosted additive model tool Maaslin. Bacterial relative abundance means across each category shown as the size and significance as the shade of each *circle* (*darker* = smaller *p* value; Additional file 2: Table S1). Significant association of the microbiome with the continuous variables weight gain or age is shown with symbols (positively (*+*) or negatively (*−*) associated; Additional file 2: Table S1). Genera sorted taxonomically with subgroups within the *Firmicutes* labeled in *grey*

Difference in the relative abundance of the dominant bacterial genera is presented as a taxa bar chart in Additional file 1: Figure S2, broken down by ethnic group and breastfeeding status. The relative abundance of individual bacterial genera was assessed for association with ethnicity and breastfeeding while accounting for infant age and weight gain in the first year. These covariates, which had survived the stepwise regression on the entire community, were included in the multivariate algorithm in order to strike a balance between overfitting the model and identifying the most comprehensive list of predictors. Taxa significantly associated with ethnicity, breastfeeding at time of collection, infant weight gain in the first year, and infant age (q value <0.05) are listed in Additional file 2: Table S1 and their abundance is illustrated in Fig. 3.

South Asians had higher abundances of several genera within the *Actinobacteria* (*Bifidobacterium*, *Collinsella*, *Actinomyces*, *Atopobium*) and of three unclassified genera compared to white Caucasians. Genera within the phylum *Firmicutes* within two distinct taxonomic groups were associated with ethnicity. Genera such as *Streptococcus*, *Enterococcus*, and *Lactobacillus* (class *Bacilli*, order *Lactobacillales*) were more abundant within South Asians whereas genera such as *Blautia*, *Pseudobutyrivibrio*, *Ruminococcus*, and *Oscillospira* (order *Clostridiales*) were more abundant in white Caucasians*.* The most differentially abundant genus were unclassified members of the *Lachnospiraceae* which were higher in white Caucasians. In order to investigate whether these differences were specific to each cohort or were indicative of true ethnic differences, five genera significantly associated with either white Caucasians or South Asians were plotted among the small number of South Asians recruited within the CHILD cohort (n = 6 that were not used for the previous microbiome analysis). Despite the small number available, the same trends were seen for the five genera plotted (Additional file 1: Figure S5).

Not surprisingly, breastfeeding status at the time of sample collection was strongly associated with the abundance of the genera *Bifdobacterium* (phylum *Actinobacteria*; Fig. 3). Several genera within the phylum *Firmicutes* were associated with breastfeeding at the time of collection; some were more abundant (*Veillonella*, *Megasphaera*, and *Dialister*) and others were less abundant (*Blautia*, unclassified *Lachospiraceae*, *Clostridium*, *Ruminococcus*, *Coprobacillus*, *Lactococcus*, as well as several unclassified genera within the *Clostridiales* and *Erysipelotrichales*).

## Discussion

Our results demonstrate that the gut microbiome of infants is influenced by ethnicity, infant age, weight gain, and breastfeeding. The gut microbiome has been proposed to influence the progression of chronic diseases and has been associated with adverse health outcomes [42]. Development of the microbiome within the first years of life may influence long-term health, and can be affected by perinatal, genetic, and dietary factors, including solid foods and milk diet.

The distribution of a number of maternal and infant parameters differed between white Caucasian and South Asians (i.e., vegetarian status, gestational diabetes mellitus prevalence, timing of introduction of solid foods, antibiotic use during pregnancy, mode of delivery, etc.) and thus seemed likely candidates to explain the microbiome differences by ethnicity. However, when these variables were added as independent predictors of the gut microbiome composition in the multivariable model, none except breastfeeding status at the time of sampling, infant age, and weight gain in the first year improved the fit of the model (ethnicity R^2^ = 0.084 versus R^2^ = 0.082 with all additional variables; breastfeeding status R^2^ = 0.040 versus R^2^ = 0.032 with all additional variables). This suggests that these variables were largely captured by the higher order variables of interest, i.e., ethnicity and breastfeeding. Next, after taking into account these significant predictors (breastfeeding status, infant age at 1-year stool, and weight gain in the first year of life) we found that groups of bacterial genera which are phylogenetically distinct (i.e., within the order *Lactobacillales* versus *Clostridales*) were present at different abundances within each ethnic group. This suggests that different metabolic strategies are at work within the gut microbiome of South Asian and white Caucasian infants. Additionally, these bacterial taxa are good candidates to predict diet-related influences on the microbiome, microbial influences on host metabolism, and bacterial stimulation of the host immune system [43].

Several members of the lactic acid bacteria (LAB), specifically *Bifidobacterium*, *Lactococcus*, *Streptococcus*, and *Enterococcus*, were more abundant within South Asians after taking into account breastfeeding status at the time of collection, infant age, and weight gain in the first year. LAB break down mainly carbohydrates that are not absorbed by the host to produce acetate and lactate, both of which are used as energy sources by other microbial groups [43, 44]. Also the abundance of members of the *Atopobium* cluster of *Actinobacteria* (i.e., genera such as *Collinsella* and *Atopobium*) was higher in South Asians. This group of bacteria are saccharolytic (i.e., they break down small sugars) [45] and have been seen to decrease in abundance in the microbiome of individuals with a diet rich in whole grains [46]. These genera have also been associated with higher levels of low-density lipoprotein in humans [47] and, along with other members of the *Actinobacteria*, have been associated with high hepatic levels of triglycerides and low hepatic levels of glycogen and glucose in mice [48]. It is of interest to note that these observations are based on v3 16S rRNA gene data. Several studies of the infant gut microbiome, which employ amplification and sequencing of other variable regions of the same gene often report very low levels of *Actinobacteria* [6, 9, 49]. Members of this phylum, such as the *Bifidobacteria*, have been shown to dominate the infant gut microbiome [4, 50, 51], suggesting a possible primer bias against this group.

In contrast, white Caucasians showed higher abundances of members of the *Firmicutes* from the order *Clostridiales*, which have been shown to be increased in response to diets rich in animal protein [52] and high in fat [53]. Products of bacterial fermentation of acetate and lactate, mentioned above, as well as non-digestible fiber and oligosaccharides by members of the *Clostridiales* seen here (*Ruminococcus*, *Lachnospiraceae*, and *Oscillospira*) include short chain fatty acids like butyrate, which is used by host cells as an energy source and can signal increased barrier function [43]. Though also proposed to be chemoprotective, the relationship between luminal butyrate exposure and colorectal cancer in humans has been examined only indirectly in case-control studies [54]. Nevertheless, these findings suggest different metabolic processes and immune stimuli at work within the South Asian and white Caucasian infant gastrointestinal tract, some of which may be explained by their heterogeneous diets.

When switching from a milk-based diet to a solid food diet, prior studies have shown a decrease in the abundance of *Bifidobacterium* along with an increase in members of the *Firmicutes* (such as *Clostridium* sp.) and *Bacteroidetes* [12, 20]. One study suggests that it is the cessation of breastfeeding that is required for maturation of the gut microbiota to occur with a decrease in *Bifidobacterium* and an increase in members of the *Clostridiales* only occurring after weaning [9]. As expected, after adjustment for ethnicity, infant age, and weight gain in the first year, *Bifidobacterium* and *Lactobacillus* were significantly associated with breastfeeding. Additionally, an increase in the abundance of several genera within the phylum *Firmicutes* were associated with not being breastfed at the time of sampling.

*Bifidobacteria*, along with the LAB, are known to be abundant members of the microbiome of breastfeeding infants [55], whereas genera within the order *Clostridiales* are known to be more abundant within the gut of adults [56]. Here bacterial profiles indicative of a breast milk diet were common among South Asians even those that were not breastfeeding at the time of collection, suggesting that these infants retain more of a breastfeeding microbiome than do white Caucasians of the same age. The reasons for this are unclear; however, dietary differences may be contributing. Our data show that equal proportions of infants in both groups were breastfed in the first year but does not capture breastfeeding frequency. It also shows that there was a much higher rate of formula use and an earlier introduction to solid food within South Asian than within white Caucasian infants. Because self-reported vegetarianism was more frequent in South Asians, it is possible that meat consumption hastens, or non-meat diets delay, changes induced within the infant gut microbiome during the switch to a solid food diet. It is important to note, however, that to our knowledge an analysis of the adult South Asian microbiome has not been reported, nor has a description of the maturation of the South Asian infant microbiome toward an adult-like composition; thus, our data must be interpreted within the context of the study, i.e., of South Asian infants born in Canada who consume a South Asian diet.

The underlying construct of “Ethnicity” brings together several biological and cultural factors, and it can be characterized using a number of different parameters (e.g., dietary habits, ancestral country of origin, etc.). In our multivariate model, ethnicity and breast feeding status remained independent and significant predictors of differences in the overall microbial communities (beta diversity), whereas vegetarian diet did not, which implies that the impact of ethnicity which incorporates some unique dietary patterns is not wholly explained by these dietary differences, as it also reflects other differences between the groups. After ensuring that these additional factors were potentially accounted for (i.e., years living in Canada, antibiotic use, timing of solid food introduction, etc.) we observed that breastfeeding, infant age, and weight gain in the first year significantly influenced the infant gut microbiome.

Strengths of our study include its relatively large size of nearly 200 infants from each of two different ethnic groups who have diverse dietary intakes; the availability of stool samples collected at similar times using similar methods; the high quality deep sequencing of the 16S rRNA gene for bacterial identification; a reliability analysis to demonstrate reproducibility of our methods; and detailed measurement of maternal and infant covariates. Limitations include incomplete data on maternal weight gain during and prior to pregnancy, which limits our ability to assess the influence of this important covariate on the infant gut microbiome; ethnicity in this study refers to the group a person self-identifies with and reflects a mix of cultural factors, including language, diet, religion, and ancestry—thus, ethnicity is a multidimensional construct which includes some within-group heterogeneity, and differences attributable to ethnicity may reflect a broad range of factors which are not purely biological; and the lack of a direct measure of infant dietary intake beyond feeding type at the time of stool collection.

## Conclusions

The infant gut microbiome is influenced by ethnicity and breastfeeding in the first year of life. Ethnic differences in the gut microbiome may reflect maternal/infant dietary differences and whether these differences are associated with future cardiometabolic outcomes can only be determined after prospective follow-up.

## Additional files

Additional file 1: Supplementary Figures S1–S5. (PDF 2180 kb)

Additional file 2: Supplementary Table S1. (DOCX 151 kb)

## Abbreviations

CHILD: Canadian Healthy Infant Longitudinal Development study
CVD: Cardiovascular disease
LAB: Lactic acid bacteria
NCD: Non-communicable disease
OTU: Operational taxonomic unit
START: South Asian Birth Cohort
