# Supplementary material for: Ethnic and diet-related differences in the healthy infant microbiome
Source: Genome Med. 2017 Mar 29;9:32. doi: 10.1186/s13073-017-0421-5 (PMC5372248; doi:10.1186/s13073-017-0421-5)
Supplement: Supplementary file 1 — Supplementary Figures S1–S5. (PDF 2180 kb) [file 13073_2017_421_MOESM1_ESM.pdf]

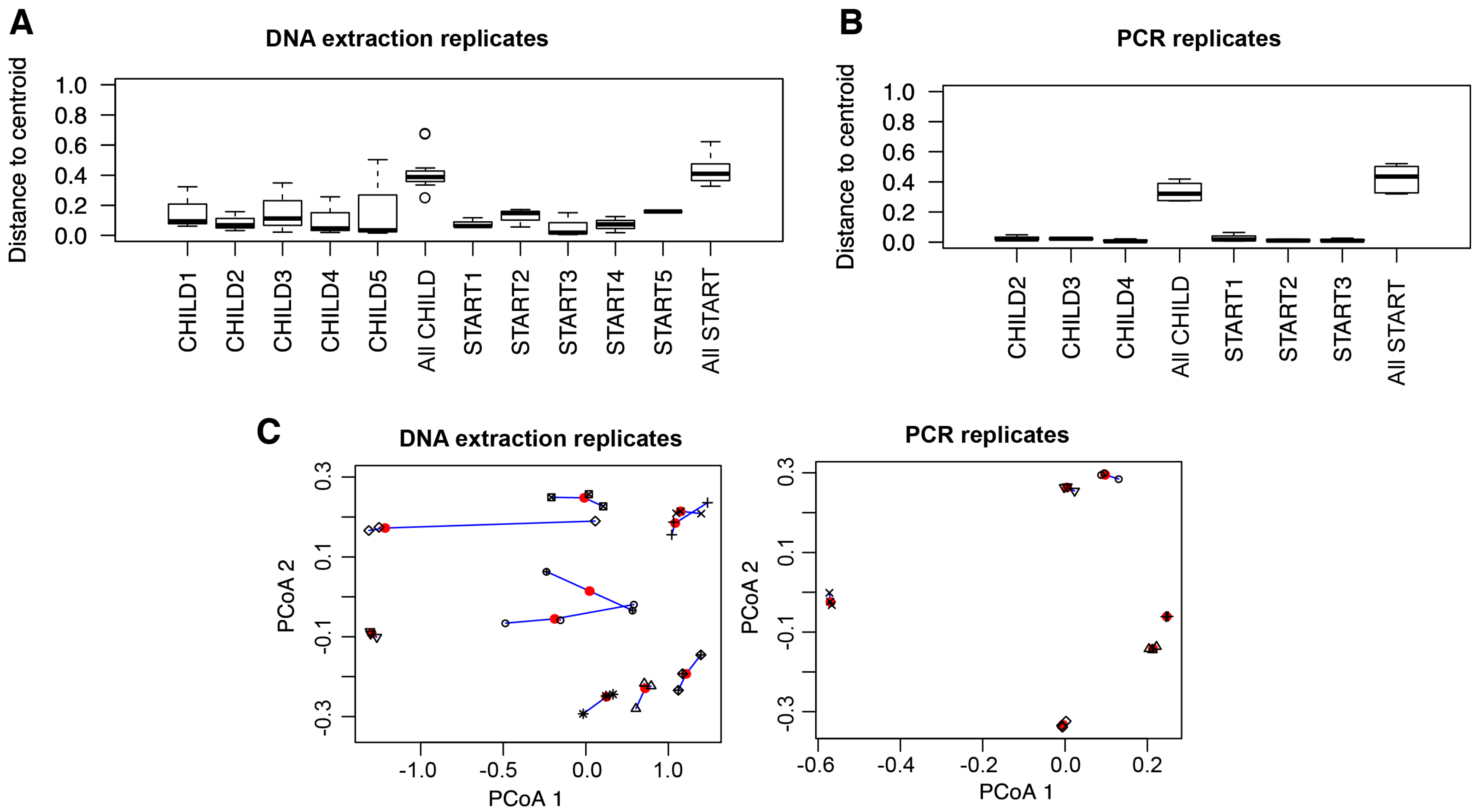

Figure S1: Bray-Curtis dissimilarity measures illustrate the reproducibility of microbiome samples. A) Dissimilarity between DNA extraction replicates for three replicates of five samples from each cohort is illustrated next to the dissimilarity between all five samples from the same cohort. B) Dissimilarities between three PCR replicates is shown next to the dissimilarity between all PCR amplifications from each cohort. C) Principal coordinate analysis illustrates reproducibility of DNA replicates and PCR replicates.

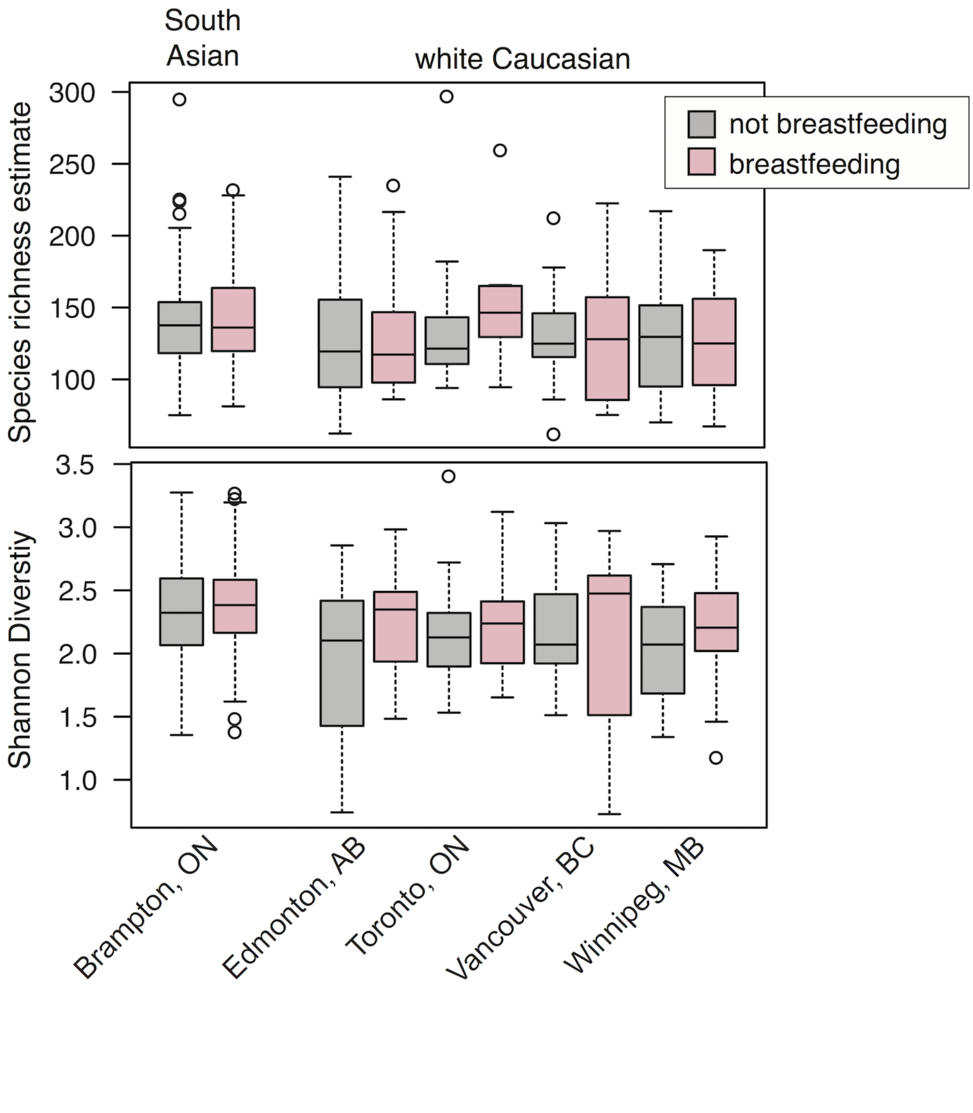

Figure S2: Alpha diversity of the gut microbiome for currently breastfeeding and not currently breastfeeding infants divided by study centre.

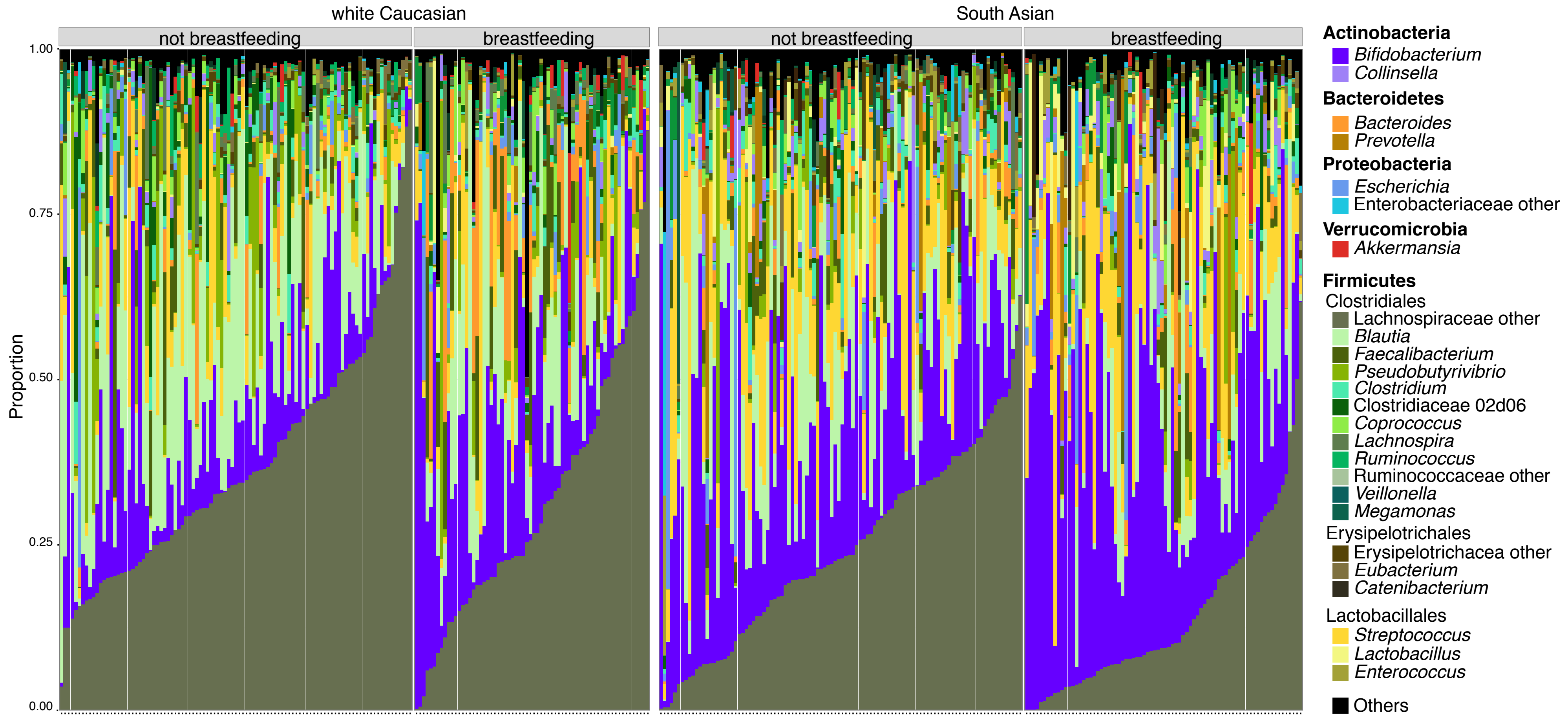

Figure S3: Ethnic and diet related differences in the microbiome of infants. Stacked bar charts illustrate differences in the abundance of different bacterial taxa between white Caucasian and South Asian infants stratified by breastfeeding status at the time of sample collection. The legend is ordered first by Phylum, then by Order for the Firmicutes, then genera are presented in order of decreasing abundance.

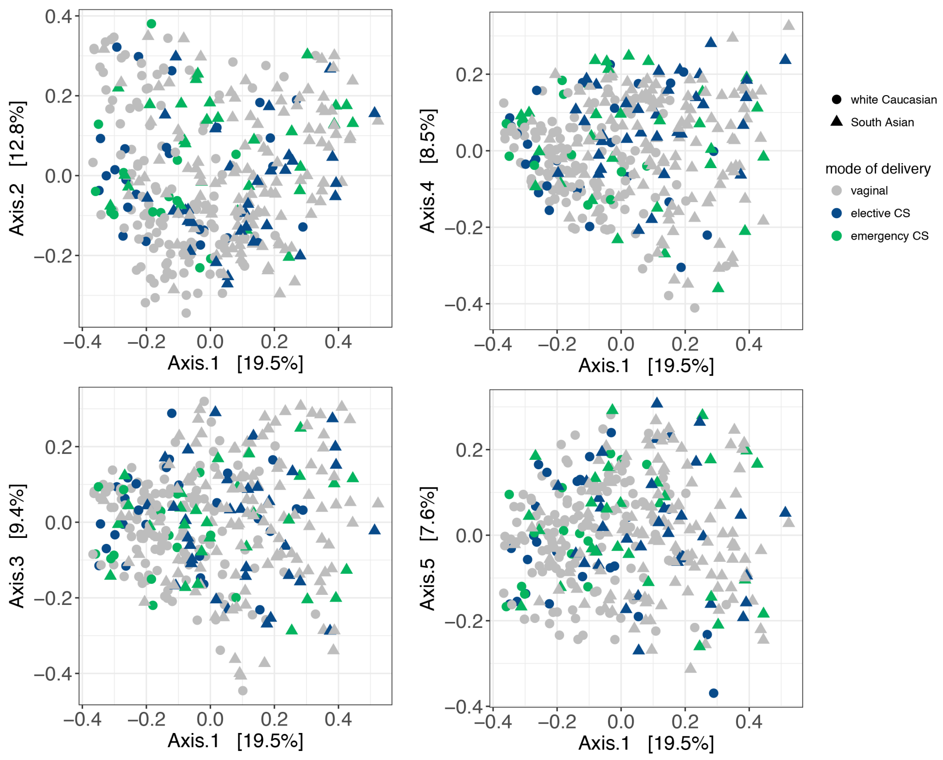

Figure S4: Delivery mode was not a significant predictor of the infant gut microbiome at one year. PCoA analysis of Bray Curtis dissimilarity, samples coloured for delivery mode, shapes indicate ethnicity. PC axes 1-5 are shown. CS = Ceasarean section.

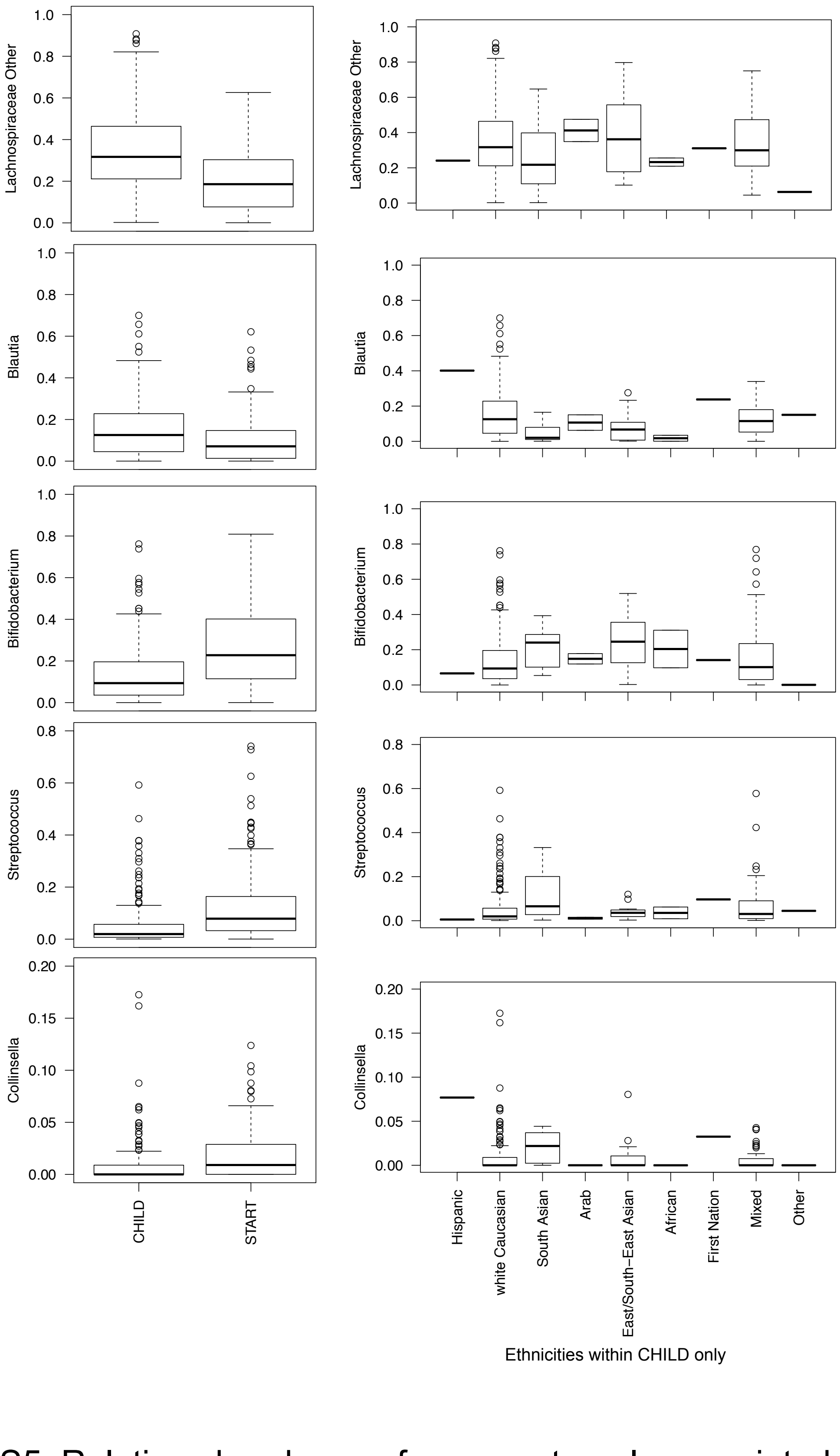

Figure S5: Relative abundance of genera strongly associated with ethnicity (CHILD vs START) in this study and the relative abundance of the same genera between different ethnicities within the CHILD cohort.
