# Supplementary material for: Ethnic and diet-related differences in the healthy infant microbiome
Source: Genome Med. 2017 Mar 29;9:32. doi: 10.1186/s13073-017-0421-5 (PMC5372248; doi:10.1186/s13073-017-0421-5)
Supplement: Supplementary file 2 — Supplementary Table S1. (DOCX 151 kb) [file 13073_2017_421_MOESM2_ESM.docx]

**Table S1: Genera differentially associated with ethnicity, infant diet, infant age or infant weight gain in the first year.**

| **Ethnicity** |  |  |  |  |
| --- | --- | --- | --- | --- |
| **Phylum Order Genus** | **Coefficient ^a^** | **N > 0** | **p value** | **q value** |
| Actinobacteria Coriobacteriales *Eggerthella* | -0.006 | 295 | 4.80E-05 | 4.90E-04 |
| Bacteroidetes Bacteroidales Rikenellaceae Other | -0.008 | 124 | 1.10E-03 | 6.50E-03 |
| Firmicutes Clostridiales Lachnospiraceae Other | -0.183 | 335 | 5.30E-16 | 1.70E-13 |
| Firmicutes Clostridiales *Blautia* | -0.074 | 335 | 8.90E-04 | 5.30E-03 |
| Firmicutes Clostridiales *Pseudobutyrivibrio* | -0.034 | 334 | 8.20E-04 | 5.00E-03 |
| Firmicutes Clostridiales 02d06 | -0.029 | 329 | 5.20E-05 | 5.10E-04 |
| Firmicutes Clostridiales *Ruminococcus* | -0.015 | 331 | 2.10E-06 | 4.20E-05 |
| Firmicutes Clostridiales *Oscillospira* | -0.012 | 318 | 5.00E-06 | 7.60E-05 |
| Proteobacteria Pseudomonadales *Pseudomonas* | -0.003 | 116 | 2.00E-11 | 1.10E-09 |
| Actinobacteria Bifidobacteriales *Bifidobacterium* | 0.17 | 335 | 2.70E-12 | 1.80E-10 |
| Actinobacteria Coriobacteriales *Collinsella* | 0.051 | 288 | 8.00E-08 | 2.30E-06 |
| Actinobacteria Actinomycetales Other | 0.012 | 231 | 5.00E-07 | 1.20E-05 |
| Actinobacteria Bifidobacteriales Bifidobacteriaceae Other | 0.012 | 245 | 2.50E-13 | 2.70E-11 |
| Actinobacteria Coriobacteriales Coriobacteriaceae Other | 0.006 | 119 | 1.00E-05 | 1.20E-04 |
| Actinobacteria Actinomycetales *Actinomyces* | 0.004 | 314 | 9.90E-07 | 2.10E-05 |
| Actinobacteria Coriobacteriales *Atopobium* | 0.003 | 160 | 1.30E-14 | 2.20E-12 |
| Bacteroidetes Bacteroidales *Prevotella* | 0.003 | 274 | 8.50E-05 | 7.70E-04 |
| Firmicutes Erysipelotrichales Erysipelotrichaceae Other | 0.003 | 268 | 6.70E-04 | 4.20E-03 |
| Firmicutes Lactobacillales *Streptococcus* | 0.11 | 335 | 4.00E-09 | 1.90E-07 |
| Firmicutes Lactobacillales *Enterococcus* | 0.029 | 311 | 5.20E-09 | 2.10E-07 |
| Firmicutes Lactobacillales *Lactobacillus* | 0.014 | 296 | 1.90E-04 | 1.30E-03 |
| Firmicutes Lactobacillales Other | 0.004 | 262 | 2.70E-05 | 3.00E-04 |
| Proteobacteria Enterobacteriales Enterobacteriaceae Other | 0.013 | 291 | 8.10E-07 | 1.90E-05 |
| Proteobacteria Enterobacteriales *Klebsiella* | 0.004 | 242 | 1.10E-03 | 6.20E-03 |
| **Breastfeeding** |  |  |  |  |
| **Phylum Order Genus** | **Coefficient ^b^** | **N > 0** | **p value** | **q value** |
| Firmicutes Clostridiales *Blautia* | -0.103 | 335 | 4.30E-06 | 7.00E-05 |
| Firmicutes Clostridiales Lachnospiraceae Other | -0.102 | 335 | 3.60E-06 | 6.50E-05 |
| Firmicutes Clostridiales *Clostridium* | -0.03 | 334 | 1.30E-04 | 1.00E-03 |
| Firmicutes Clostridiales *Ruminococcus* | -0.012 | 331 | 8.90E-05 | 7.70E-04 |
| Firmicutes Clostridiales Other | -0.007 | 326 | 6.50E-05 | 6.20E-04 |
| Firmicutes Clostridiales Peptostreptococcaceae Other | -0.003 | 278 | 3.40E-03 | 1.80E-02 |
| Firmicutes Erysipelotrichales Erysipelotrichaceae Other | -0.018 | 330 | 1.50E-03 | 8.00E-03 |
| Firmicutes Erysipelotrichales Erysipelotrichaceae Other | -0.004 | 268 | 3.60E-04 | 2.40E-03 |
| Firmicutes Erysipelotrichales *Coprobacillus* | -0.003 | 167 | 1.10E-02 | 4.80E-02 |
| Firmicutes Lactobacillales *Lactococcus* | -0.003 | 234 | 8.10E-03 | 3.80E-02 |
| Actinobacteria Bifidobacteriales *Bifidobacterium* | 0.106 | 335 | 1.00E-05 | 1.20E-04 |
| Firmicutes Clostridiales *Veillonella* | 0.02 | 327 | 3.80E-04 | 2.50E-03 |
| Firmicutes Clostridiales *Megasphaera* | 0.011 | 224 | 4.20E-08 | 1.40E-06 |
| Firmicutes Clostridiales *Dialister* | 0.003 | 194 | 6.70E-06 | 9.30E-05 |
| Firmicutes Lactobacillales *Lactobacillus* | 0.014 | 296 | 1.70E-04 | 1.30E-03 |
| **Infant age**  **Phylum Order Genus** | **Coefficient** | **N > 0** | **p value** | **q value** |
| Proteobacteria Enterobacteriales *Escherichia* | -0.008 | 331 | 0.001 | 0.007 |
| Firmicutes Clostridiales *Butyricicoccus* | 0.004 | 211 | 9.87E-06 | 1.23E-04 |
| Firmicutes Clostridiales Ruminococcaceae Other | 0.004 | 286 | 4.55E-05 | 4.76E-04 |
| Firmicutes Clostridiales *Ruminococcus* | 0.007 | 316 | 1.58E-04 | 0.001 |
| Firmicutes Clostridiales *Pseudobutyrivibrio* | 0.014 | 334 | 1.11E-04 | 0.001 |
| Firmicutes Clostridiales *Coprococcus* | 0.014 | 319 | 9.08E-05 | 0.001 |
| **Infant weight gain in the first year**  **Phylum Order Genus** | **Coefficient** | **N > 0** | **p value** | **q value** |
| Bacteroidetes Bacteroidales Bacteroides | -0.015 | 332 | 0.0003 | 0.0019 |
| Firmicutes Clostridiale Ruminococcus | -0.006 | 316 | 0.0054 | 0.0275 |
| Firmicutes Clostridiales Ruminococcaceae Other | -0.003 | 286 | 0.0094 | 0.0431 |
| Actinobacteria Other | 0.001 | 272 | 0.0059 | 0.0295 |

^a^ Coefficient for SA compared to WC; ^b^ currently breastfed compared to not currently breastfed.
